# Study rationale and design of a study of EMPAgliflozin’s effects in patients with type 2 diabetes mellitus and Coronary ARtery disease: the EMPA-CARD randomized controlled trial

**DOI:** 10.1186/s12872-021-02131-1

**Published:** 2021-06-30

**Authors:** Sepehr Gohari, Tara Reshadmanesh, Hadi Khodabandehloo, Mojtaba Fathi, Hassan Ahangar, Shahram Arsang-Jang, Faramarz Ismail-Beigi, Samin Ghanbari, Mohsen Dadashi, Muhammad Javad Muhammadi, Sheida Gohari, Saeid Ghaffari

**Affiliations:** 1 Student Research Center, School of Medicine, Zanjan University of Medical Sciences, Zanjan, Iran; 2 Department of Clinical Biochemistry, School of Medicine, Zanjan University of Medical Sciences, Zanjan, Iran; 3 Department of Cardiology, Mousavi Hospital, School of Medicine, Zanjan University of Medical Sciences, Zanjan, Iran; 4 Department of Biostatistics, School of Medicine, Zanjan University of Medical Sciences, Zanjan, Iran; 5 Department of Medicine, Case Western Reserve University, University Hospitals Cleveland Medical Center, Cleveland, OH USA; 6 Department of Systems Science and Industrial Engineering, State University of New York at Binghamton, Binghamton, NY USA

**Keywords:** SGLT2 inhibitor, Empagliflozin, Randomized controlled trial, Coronary artery disease, Inflammation, Type 2 diabetes mellitus

## Abstract

**Background:**

Recent trials have revealed that sodium-glucose co-transporter 2 inhibitors (SGLT2-i) are effective against hyperglycemia and also reduce micro- and macro-vascular complications in patients with type 2 diabetes mellitus (T2DM). Most of the beneficial cardiovascular effects have been investigated in patients with heart failure and coronary artery disease (CAD). Yet, few human studies have been conducted to investigate the molecular mechanisms underlying these clinically beneficial effects in patients with CAD. Accordingly, the EMPA-CARD trial was designed to focus on the molecular effects of empagliflozin in patients with T2DM and CAD.

**Methods:**

In this multicenter, triple-blind randomized controlled trial, patients with documented known T2DM and CAD will be recruited. They will be randomized on a 1:1 ratio and assigned into two groups of empagliflozin 10 mg/daily and placebo. The primary endpoint is the effect of empagliflozin on changes of plasma interleukin 6 (IL-6) after 26 weeks of treatment. The secondary endpoints will consist of changes in other inflammatory biomarkers (Interleukin 1-beta and high-sensitive C-reactive protein), markers of oxidative stress, platelet function, and glycemic status.

**Discussion:**

The EMPA-CARD trial mainly tests the hypothesis that SGLT2 inhibition by empagliflozin may improve inflammatory status measured as reduction in inflammatory biomarkers in patients with T2DM and CAD. The results will provide information about the underlying mechanisms of SGLT2 inhibition that mediate the beneficial effects of this medication on clinical outcomes.

***Trial registration*:**

Iranian Registry of Clinical Trials. www.IRCT.ir, Identifier: IRCT20190412043247N2. Registration Date: 6/13/2020. Registration timing: prospective.

**Supplementary Information:**

The online version contains supplementary material available at 10.1186/s12872-021-02131-1.

## Background

Diabetes mellitus (DM) is a chronic disease that is characterized by high blood glucose levels due to the inability to produce adequate amounts of insulin and/or resistance to the hormone’s actions [1]. Type 2 diabetes mellitus (T2DM) is a complex metabolic condition and, as the most common type of diabetes, it affects some 37% of the population in western and middle-eastern societies leading to long-term complications [2]. The major complications of this disease fall into two general categories of microvascular and macrovascular involvements, which can consequently lead to cardiovascular, neurological, ophthalmological, and renal damage [3]. Cardiovascular complications are the leading cause of death in the affected population. Atherosclerosis develops more rapidly in these patients and increases the incidence of a heart attack two to three times [4]. The management methods require multi-faceted strategies, including regular physical activity, proper diet, and medications to control hyperglycemia, hypertension, inflammation, and dyslipedemia [5, 6]. Among various medications available to treat diabetes, one of the newest ones is sodium-glucose co-transport 2 inhibitors (SGLT2-i) including empagliflozin, dapagliflozin, and canagliflozin. Empagliflozin is a selective inhibitor of the SGLT2 co-transporter expressed in the proximal tubule of nephrons. Inhibition of SGLT2 reduces hyperglycemia by decreasing glucose reabsorption in the kidneys leading to increased urinary glucose excretion, reductions in blood glucose and HbA_1C_ [7]. The EMPA-REG OUTCOME trial and its sub-studies revealed the additional beneficial effects of this medication versus standard treatment protocols in patients with T2DM and heart failure. These effects include a reduction in Major Adverse Cardiovascular Events (MACE), blood pressure, urinary albumin exertion, and improvements in lipid profile and vascular resistance [8, 9]. Some hypotheses have been advanced to explain the beneficial cardiovascular effects of this agent including hemodynamic changes due to decreased plasma volume following glycosuria-induced diuresis, changes in heart fuel consumption, improvements in overall metabolism by a reduction of inflammation, and direct effects on the cardiac tissue; however, the underlying mechanisms remain obscure [10, 11]. Many factors contribute to the physiopathology of cardiovascular complications in patients with T2DM including an increase in pro-inflammatory factors, imbalance in oxidative stress and a hyper-coagulation state with abnormal platelet function [12]. In this regard, animal models and in-vitro studies have been performed to evaluate the effects of empagliflozin on inflammatory state and platelet function. The results showed a reduction in oxidative stress, pro-inflammatory phenotype, and glucotoxicity, but platelet function remained unchanged [13, 14]. To date, few human model randomized trial has been performed to evaluate the inflammation and platelet dysfunction hypothesis in T2DM patients with coronary artery disease (CAD). Hence, the main objective of this parallel group, triple-blind randomized placebo-controlled trial is to test the hypothesis that empagliflozin improves the inflammatory state in T2DM patients with documented known CAD.

## Methods and experimental design

### Study design and Intervention

EMPA-CARD is a multicenter triple-blind randomized placebo-controlled trial designed to evaluate the effect of treatment with empagliflozin (10 mg tablet once daily) versus placebo for 26 weeks, in addition to standard care, in patients with T2DM and coronary artery disease.

#### Study objectives

**Fig. 1:**
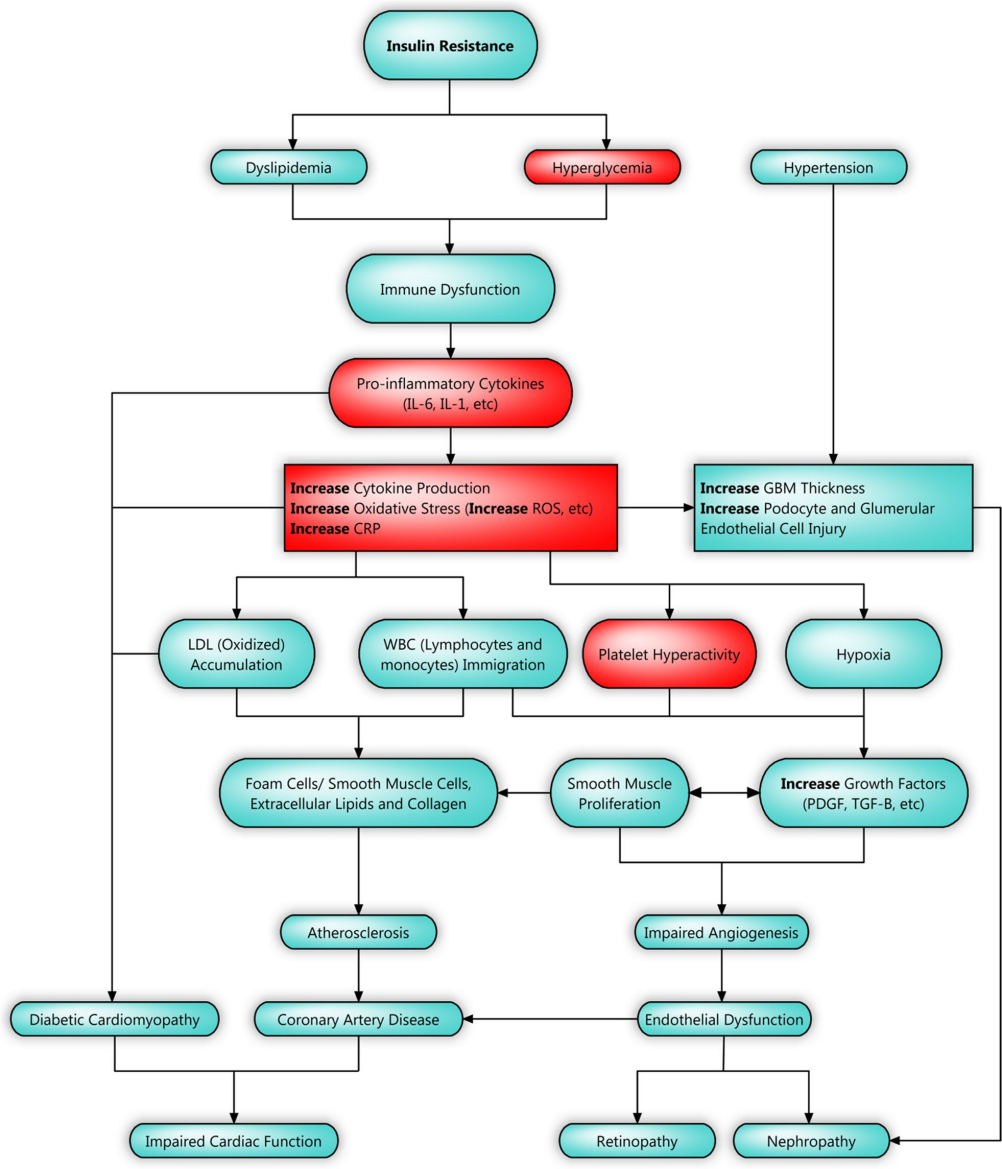
Pathways contributing to the development of micro- and macro-vascular complications in patients with T2DM and insulin resistance (only coronary artery disease, retinopathy and nephropathy are illustrated). Red boxes Indicate where the trial’s primary objective (potential molecular effects of empagliflozin on inflammatory state) will be assessed. The concept was derived from the studies of Bugger et al. and Oguntibeju et al. [15, 16]. *IL-6* interleukin 6, *IL-1* interleukin 1, *ROS* reactive oxygen species, *CRP* C-reactive protein, *GBM* glomerular basement membrane, *LDL* low density lipoprotein, *WBC* white blood cell, *PDGF* platelet derived growth factor, *TGF-β* transforming growth factor beta

The primary objective of the EMPA-CARD trial is to evaluate the effect of empagliflozin on the inflammatory state in patients with T2DM and coronary artery disease after 26 weeks of treatment (Fig. 1). Other objectives which will be investigated as the trial’s sub-studies are shown in Table 1.

**Table 1:** Trial objectives and measurement methods (measured at time = 0 and after 26 weeks of treatment)

Objective	Definition
Primary	1. To investigate the impact of empagliflozin on plasma IL-6 level in patients with T2DM patients and coronary artery disease (established by coronary angiography)
Secondary	1. Inflammatory biomarkers Changes in serum hs-CRP levels Changes in plasma IL-1b levels 2. Oxidative stress status Changes in lymphocytic reactive oxygen species Changes in plasma levels of malondialdehyde Changes in plasma carbonyl levels Changes in plasma antioxidant capacity Changes in plasma reduced glutathione levels Changes in catalase enzyme activity Changes in plasma superoxide dismutase enzyme activity 3. Platelet function Changes in CD62-P expression on the platelet surface 4. Glycemic status Changes in fasting blood glucose levels Changes in HbA_1c_ levels Changes in HOMA-IR
Additional secondary (Considered for sub-studies)	1. Changes is cardiac biomarkers Changes in serum hs-Troponin I levels Changes in serum BNP levels Changes in serum NT-proBNP levels 2. Echocardiographic parameters Changes in left ventricular systolic function Changes in left ventricular diastolic function Changes in right ventricular function 3. Hematopoietic status and renal function Changes in serum erythropoietin levels Changes in blood hematocrit levels Changes in hemoglobin levels Changes in urine micro albuminuria levels 4. Changes in lipid profile Changes in serum total cholesterol levels Changes in serum LDL cholesterol levels Changes in serum HDL cholesterol levels Changes in serum triglyceride levels 5. Electrocardiographic parameters Changes in PR interval duration Changes in QRS complex duration Changes in QT interval duration Changes in ST segment deviation Changes in T wave alternans

*IL-6* Interleukin 6, *T2DM* type 2 Diabetes mellitus, *hs-CRP* high sensitive, *CRP* C-reactive protein, *IL-1b* interleukin 1-beta, *HbA*_*1c*_ glycated hemoglobin A_1c_, *HOMA-IR* Homeostatic Model Assessment for Insulin Resistance, *hs-Troponin I* high sensitive troponin I, *BNP* brain natriuretic peptide, *NT-proBNP* N-terminal pro-brain natriuretic peptide, *LDL cholesterol* low density lipoprotein cholesterol, *HDL cholesterol* high density lipoprotein cholesterol

#### Ethics considerations

The EMPA-CARD trial is conducting in accordance with the principles of the declaration of Helsinki and all subsequent revisions. The study was approved by the ethics committee of Zanjan university of Medical Sciences. All patients are provided with written informed consent by the investigators prior to the recruitment. Moreover, the study protocol was prospectively registered on the Iranian Registry of Clinical Trials (www.IRCT.ir, Identifier: IRCT20190412043247N2).

#### Trial population and eligibility assessment

Our goal is to randomize 100 participants from registries of 4 clinical centers (*i.e.,* Mousavi hospital (coronary angiography registry), Vali-e-Asr hospital, and two cardiology clinics of Zanjan University of Medical Sciences) in Zanjan, Iran. The pooled population of the clinics is 8000 patients, whose records were extracted from registries at the pre-recruitment phase. Patients aged between 45 and 75 years old with T2DM and known coronary artery disease (established by coronary angiography) who are on background standard anti-ischemic and anti-diabetic therapy with an HbA_1C_ between 6.5 and 9.5% were eligible for inclusion. The type and dose of background anti-diabetic medications should be constant for at least 3 months prior to recruitment. Patients’ left ventricular ejection fraction (LVEF) must be higher than 40%. Moreover, they must not be receiving anti-oxidant, anticoagulant, or antiplatelet medications or supplements (except for aspirin at 80 mg/d) for at least 3 months prior to recruitment. Detailed information for inclusion and exclusion criteria is provided in Table 2.

**Table 2:** List of inclusion and exclusion criteria for randomization

Inclusion criteria	1. Age between 40 and 75 years 2. HbA1c between 6.5 and 9.5% 3. Documented known diabetes mellitus type 2 under continuous fixed-dose anti-diabetic treatment for at least 3 months prior to the randomization 4. Documented known coronary artery disease established by coronary angiography under continuous fixed-dose of anti-ischemic treatment for at least 3 months prior to the randomization 5. BMI less than 40 kg/m^2^ 6. Fixed diet and physical activity 7. eGFR greater than 45 ml/min/1.73m^2^ 8. Informed consent given in written form 9. Resting heart rate between 60 to 100 b/min
Exclusion criteria	1. Pregnancy 2. Heart Failure (NYHA class 3–4) 3. Left ventricular ejection fraction < 40% 4. Use of anti-coagulant or anti-platelet drugs (except aspirin 80 mg/daily) for at least 3 months prior to the blood sampling 5. Consumption of alcohol, continuous anti-inflammatory drugs (except aspirin 80 mg/daily) or anti-oxidant supplement 6. Use of pioglitazone 7. History of allergic reaction to SGLT2-i medications 8. History of SGLT2-i medication usage 9. Gastrointestinal malabsorption disease 10. History of CABG, ACS, TIA, CVA or PCI during past 3 month 11. History or presence of malignancy 12. Severe HTN Resting systolic blood pressure ≥ 180 mmHg and/or Resting diastolic blood pressure ≥ 120 mmHg 13. Anemia (Hb < 10 g/dl) 14. Platelet count < 100,000/µl 15. History of infection during 1 one month prior to blood sampling 16. History of heart or lung transplant 17. Major psychiatric disorders 18. History of DKA 19. Elevated liver enzymes > 3 times upper normal limit 20. Use of drugs which prolong QT interval 21. Presence of arrhythmia 22. Electrolyte imbalance 23. Patients with pace maker

*HbA*_*1c*_ glycated hemoglobin A_1c_, *BMI* body mass index, *eGFR* estimated glomerular filtration rate, *NYHA* New York Heart Association, *SGLT2-I* sodium-glucose co-transporter-2 inhibitor, *CABG* coronary artery bypass graft, *ACS* acute coronary syndrome, *TIA* transient ischemic attack, *CVA* cerebrovascular accident, *PCI* percutaneous coronary intervention, *HTN* hypertension, *Hb* hemoglobin, *DKA* diabetic ketoacidosis

#### Randomization and follow-up

The randomization method employed in this study is stratified randomization. During the screening process, eligible patients are stratified by gender (male and female), age (45–54, 55–64, and 65–75 years old), and HbA_1c_ (6.5–7.9% and 8–9.5%) and assigned into one of the two arms of the study (A or B). The randomization sequence is created using Winpepi software (version 11.6). The allocation sequence was concealed using the sealed envelopes mechanism. All participants will be followed up by phone calls every 4 weeks for assuring the proper medication usage, and for premature discontinuation or development of any adverse events. At the end of weeks 2, 12, and 26, patients are instructed to attend clinic visits for complete checkup and physical examination. Patients are also advised to return all used and unused medications at the end of week 26. The study’s follow-up and procedures are summarized in Table 3.

**Table 3:**
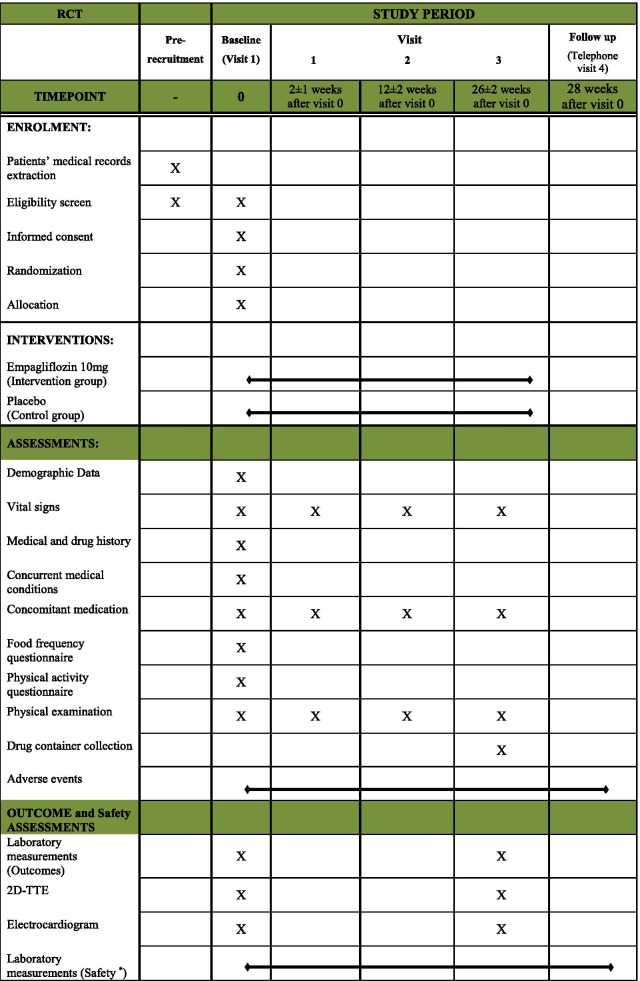
SPIRIT flow diagram

*2D-TTE* 2-dimentional trans-thoracic echocardiography

*The safety measurements were done at the visit 0, visit 3 and whenever an adverse event occurs in a patient. The measurements included liver and renal function related laboratory measurements (aspartate-aminotransferase [AST], alanine-aminotransferase [ALT], alkaline phosphatase [ALP], creatinine [Cr] and blood urea nitrogen [BUN]), serum electrolyte measurements (sodium [Na], potassium [K]), coagulative measurements (prothrombin time [PT], partial thromboplastin time [PTT]), complete blood count (CBC), urine analysis (U/A), and urine culture (U/C) if the U/A become active and/or urogenital symptoms develop

#### Blinding

Empagliflozin and placebo tablets with the same color, shape, and package with the different code combination numbers are used for groups A and B. After enrollment, a code is assigned to each patient and will be used until the end of this study. Patients, researchers and healthcare providers who gather information and assess the outcomes and analyze the data of the study are blinded to the assigned treatment (empagliflozin or placebo) such that all patients will be evaluated under the unique assigned code and the treatment groups of A or B. In case of a need for un-blinding, the research council of Zanjan University of Medical Sciences will be notified by the chief investigator to discuss the terms of un-blinding. The un-blinded treatment list is held by the Zanjan University Medical Documentation Council which generated the allocation sequence and is not involved in the study. If the un-blinding is approved by the research council, the Documentation Council will be notified and ultimately the patient will be sent to an external clinical visit for further assessment and follow-up; meanwhile the chief investigator and research council will remain blinded.

### Outcomes

To assess the outcomes of the study and sub-studies, patients undergo blood sampling, electrocardiography (ECG), and 2D trans-thoracic echocardiography (Table 3).

#### Primary outcome

The primary outcome of this study is the changes in plasma interleukin 6 levels after 26 weeks of the treatment between the two study arms.

#### Secondary outcomes for the primary objective

The secondary outcomes for testing the hypothesis of the study’s primary objective are categorized into 4 divisions including (1) Changes in additional plasma inflammatory biomarker levels (high-sensitive C-reactive protein (hs-CRP) and plasma interleukin 1-beta (IL-1b)); (2) Changes in oxidative stress status (lymphocytic reactive oxygen species levels (ROS), plasma levels of malondialdehyde (MDA), carbonyl level, total antioxidant capacity (T-AOC), reduced glutathione level (GSHr), catalase enzyme (CAT) activity, and superoxide dismutase enzyme (SOD) activity); (3) Changes in platelet function (CD62-P expression on the platelet surface); and 4) Changes in glycemic status (Fasting Blood Glucose (FBS), HbA_1C_, and Homeostatic Model Assessment for Insulin Resistance (HOMA-IR)).

#### Additional secondary outcomes

The additional secondary outcomes will be considered for the EMPA-CARD sub-studies including (1) Changes in cardiac biomarkers (Serum high-sensitive troponin I (hs-Troponin-I), brain natriuretic peptide (BNP) and N-terminal pro-brain natriuretic peptide (NT-proBNP) levels); (2) Changes in Echocardiographic parameters (left ventricular systolic and diastolic function and right ventricular function); (3) Changes in hematopoietic status and renal function (blood hematocrit, hemoglobin, and serum erythropoietin, and urine microalbuminuria levels); (4) Changes in lipid profile (serum total cholesterol, Low-Density Lipoprotein (LDL) cholesterol, High-Density Lipoprotein (HDL) cholesterol, and triglyceride levels); and (5) Changes in electrocardiographic parameters (PR interval duration, QRS complex duration, QT interval duration, ST-segment deviation, and T wave alternans).

All the above secondary outcomes will be assessed as the changes from baseline to week 26 of treatment in each study arm. Measurements of the biomarkers will be performed in the biotechnology laboratory of the Zanjan University of Medical Sciences and the Mousavi Hospital’s laboratory. The detailed information about the mentioned outcomes is presented in Table 4.

**Table 4:** The detailed descriptions of selected outcomes

Outcome	Method of measurement	Mechanism	Time of measurement
Changes in plasma IL-6 and 1-beta levels	Plasma IL-6 and IL-1b Elisa kits	Key cytokines in development and maintenance of inflammation	Week 0 and 26
Changes in oxidative stress (Lymphocytic ROS, plasma MDA, Carbonyl, T-AOC, GSHr, CAT activity and SOD enzyme activity levels)	Flow cytometry (DCFDA as dye)^a^, spectrophotometry, colorimetric and FRAP assays	ROS, MDA and carbonyl increase in inflammation and causes cell damage GSHr, AOC, CAT activity and SOD activity increase in inflammation and reduce oxidative stress	Week 0 and 26
Changes in serum hs-CRP level	Turbidimetric assay	Circulating concentrations rise in response to inflammation	Week 0 and 26
Changes in platelet function^b^	Flow cytometry (MFI of CD62-P expression on platelet surface)	Platelet activity increases in DM and inflammation causing platelet dysfunction	Week 0 and 26
Changes in HbA_1c_, FBS and Basal Insulin level	Enzymatic assay	Indicate the glycemic status which is reduce with anti-glycemic drugs	Week 0 and 26
Changes in HOMA-IR^c^	fasting insulin (mU/mL) × fasting glucose (mg/dL)/405	A method used to quantify insulin resistance which increases in DM and insulin resistance conditions	Week 0 and 26
Changes in serum hs-troponin I, BNP and NT-proBNP levels	Chemiluminescence assay	Specific cardiac biomarkers increase in cardiomyocyte damage and tension	Week 0 and 26
Changes in blood Hb, Hct and serum erythropoietin	CBC and serum erythropoietin Elisa kit	Reflects the hematopoietic status	Week 0 and 26
Changes in urine micro-albuminuria and albumin to creatinine ratio	Photometry and enzymatic assays	Reflects the renal and glomerular function. May increase in in DM as a microvascular adverse event	Week 0 and 26
Changes in lipid profile (serum TG, cholesterol, HDL,LDL cholesterols)	Enzymatic assay	Lipid disorders increase in DM which raises the cardiovascular complications that may decrease with empagliflozin treatment	Week 0 and 26
Changes in echocardiography parameters	2D trans-thoracic Echocardiography	Reflects left ventricular systolic and diastolic and right ventricular function	Week 0 and 26
Changes in electrocardiographic parameters	Electrocardiography	Reflects the electrophysiological activity of the heart	Week 0 and 26

*IL-6* interleukin 6, *IL-1b* interleukin 1-beta, *ROS* reactive oxygen species, *MDA* malondialdehyde, *T-AOC* total antioxidant capacity, *GSHr* reduced glutathione, *CAT* catalase, *SOD* superoxide dismutase, *DCFDA* dichlorofluorescin diacetate, *FRAP* fluorescence recovery after photobleaching, *hs-CRP* high sensitive C-reactive protein, *MFI* mean fluorescent index, *DM* diabetes mellitus, *HbA*_*1c*_ glycated hemoglobin, *FBS* fasting blood glucose, *HOMA-IR* homeostatic model assessment for insulin resistance, *hs-Troponin I* high sensitive troponin I, *BNP* brain natriuretic peptide, *NT-proBNP* N-terminal pro-brain natriuretic peptide, *Hb* hemoglobin, *Hct* hematocrit, *CBC* complete blood count, *TG* triglyceride, *HDL* high density lipoprotein, *LDL* low density lipoprotein cholesterol

^a^To assess the changes in ROS production, peripheral blood mononuclear cells (PBMC) will be separated from fresh whole blood and lymphocytes will be gated through sample acquisition in flow cytometric analysis

^b^To minimize the time interval between sample drawing and sample acquisition (less than 90 min) and any further manipulation which may result in unwanted platelet activation, the activation test was set up on whole blood via Thrombin Receptor Activator Peptide 6 (TRAP-6). The platelet concentrated area will be gated during sample acquisition and the MFI in histogram plot will be measured by Human CD62-P FITC antibody

^c^Patients who are under treatment with insulin in any form will be excluded from the analysis

### Biobank

For the measurements and analysis of some outcomes, a biobank is established in which the separated plasma/serum and urine samples are allocated in separate micro-tubes and stored in an ultra-low temperature freezer with the patient’s dedicated code. Based on the regulations of the local data protection agency, after the measurements, all the remaining samples will be anonymized and the biobank will be discontinued. All patients are informed in the consent form about the establishment of the biobank.

### Study monitoring

Monitoring the study and data source verification is the responsibility of the Research Council of Zanjan University of Medical Sciences, Iran which is independent from the study sponsors and any potential competing interests. All the forms used in this study, including the consent form, trial procedure manual, trial violation form, adverse events form, and outcomes’ related checklists, were evaluated and validated by a team of clinical trial monitors and the above Council. Recruitment visits are conducted under direct observation of the team. Human subject de-identification is an issue which is considered as local/regional and national requirements among all presentations and publications. To fulfill that requirement, appropriate measurements such as encoding or deletion are enforced. All of the study investigators are informed to follow the trial procedure manual. Also, any deviation from the protocol by any investigator will be recorded into the trial violation form designed by the Ethics Committee of the Zanjan University of Medical Sciences. All of the study forms are in the Persian language.

### Adverse event recording

Any adverse events, related or unrelated, whether reported by the patient or discovered by the investigators during the study follow-up (until week 28) will be recorded in the adverse events form. At the same time, the Research Council of the Zanjan University of Medical Sciences will be notified at the time of the recording. The severity and the importance of the adverse events for further decisions (e.g. discontinuation, unblinding, etc.) will be evaluated by a physician. In case of occurrence of any adverse event, patient/s will be completely followed up by the assigned physician/s and the treatment charges will be fully covered by the trial team. The standard adverse event form is presented in the Additional file 1.

### Potential confounding factors

According to the nature of the variables in primary and secondary outcomes, two potential major study confounding factors were predicted: (1) patients’ nutritional status and (2) patients’ physical activity status. To evaluate the impact of these possible confounding factors, the food frequency questionnaire (FFQ) and the physical activity questionnaire (FAQ) are used. The forms are filled at the day of recruitment. Also, patients are advised not to change their routine diet or physical activity during the study.

### Statistical considerations

#### Sample size calculations

The sample size was estimated based on the changes in plasma IL-6 level as a primary outcome of treatment. According to a previous study, the SGLT2-i dapagliflozin reduced the mean plasma IL-6 level from 6.6 to 5.8 (pg/mL) with the standard deviation (SD) of 8.9 [17]. The minimal important difference (MID) of the IL-6 level was calculated 4.45 (pg/mL) using 0.5 × SD (the distributional-based method) [18]. With a study power of 80%, an alpha level of 0.05%, and MID of 4.45, the required sample size was calculated to be 41 patients in each arm. Considering a 20% dropout during the study, the sample size was rounded up to 50 participants in each arm. The sample size was calculated using the G-power software (version 3.1.9.2).

#### Statistical analysis plan

We will use the means ± standard deviations (SD) and frequencies and percentages n (%) to interpret the results. The noninferiority margin and 95% confidence interval (CI) will be estimated for primary outcomes. The noninferiority will be accepted if the upper 95% CI for the relative risk (RR) lies below the margin of noninferiority. An intention-to-treat analysis will be used to examine the effect of missing data. Superiority analysis will be performed at α = 0.05 level. The interaction effects will be examined using graphical and statistical tests. The Kolmogorov–Smirnov test will be used to determine the normality of the distribution of continuous quantitative data. Independent T-test will be used (on condition of normal distribution) for statistical analysis of quantitatively continuous variables findings related to primary and secondary outcome including interleukin 6 levels, oxidative stress biomarkers, platelet function, glycemic status, cardiac biomarkers, echocardiographic finding, hematopoietic and renal biomarker, lipid profile and electrocardiographic parameters. The Mann–Whitney U test will be used on the condition of abnormal distribution. Chi-square test will be used to analyze qualitative data such as ST segment deviation and T-wave changes in electrocardiogram. We will consider the probability of 0.05 for the main analysis. We will perform Bayesian analysis of the covariance test to examine the effects of intervention on IL-6. The primary outcome is considered as confirmatory and the secondary outcomes are considered as exploratory. All statistical tests will be carried out in the in Rv.4 environment. The primary data of the FFQ will be evaluated by using the Nutritionist software version 4 (N4).

### Current status

At present, the mentioned sites are actively recruiting patients. The pre-recruitment phase (evaluation and extraction of 8000 patients’ medical records from hospitals and clinics registries) was started on November 11, 2019. The patients’ recruitment phase (the first patient who was consented and randomized) was started on June 29, 2020 and until November 9, 2020, 91 patients were randomized. The end date of expected recruitment may be extended due to the COVID-19 pandemic but it is expected that the recruitment phase will ends by the end of January 2021.

## Discussion

The EMPA-CARD randomized placebo-controlled clinical trial seeks to investigate the mechanisms underlying the cardiovascular effects of empagliflozin in patients with T2DM who have proven CAD at the same time.

Most of the recent published clinical trials have focused on the effects of empagliflozin in patients with heart failure in which the superiority of empagliflozin has been reported. The recent meta-analysis with the pooled population of 8474 patients (DAPA-HF and EMPEROR-Reduced population) showed that treatment with an SGLT2-i was associated with a significant reduction in cardiovascular death (pooled hazard ratio of 0.86) and re-hospitalization due to heart failure complications as well as improvements in renal function [19]. However, limited data are available regarding the beneficial effects of empagliflozin in patients with established CAD. The EMPA-HEART Cardiolink-6 trial showed that 6 months of treatment with empagliflozin significantly reduced left ventricular mass index (LVMi) in patients with T2DM and CAD [20]. In this study we are exploring molecular mechanisms and cellular changes that potentially mediate these beneficial effects. Plasma levels of IL-6, a pro-inflammatory cytokine which also has anti-inflammatory properties promotes and regulates inflammatory processes in patients with T2DM, and changes in its level is considered as the primary outcome of this study. The rationale of choosing this biomarker as a leading factor for investigation among other candidates is that IL-6 and tumor necrosis factor-alpha (TNF-α) appear to have the highest impact on the promotion of CAD in patients with diabetes mellitus [21]. Moreover, the other factors measured in this study are either directly or indirectly affected by IL-6. It has been shown that IL-6 reduces oxidative stress and apoptosis of pancreatic β-cell by promoting autophagy, an adaptive response to cellular stress. Previous human studies have shown that concomitant treatment with empagliflozin and metformin in patients with type 1 diabetes reduced IL-6 up to 1.34 fold and improved anti-oxidative status parameters comprising of total anti-oxidative status (TAS) for up to 1.07 fold and superoxide dismutase (SOD) for up to 1.08 fold. It has also been suggested that the cardio-protective effects of this drug class may be associated with its anti-oxidative effects, including normalizing interferon-γ (IFN-γ), TNF-α and IL-6 levels especially with empagliflozin [22, 23]. In animal studies, diabetic rats which were treated by SGLT2 inhibitor had a lower development of endothelial dysfunction, oxidative stress, advanced glycation end products/ receptor for advanced glycation end products (AGE/RAGE) signaling and inflammation due to inhibition of the activity of NADPH oxidase and decreased serum levels of the AGE precursor methylglyoxal [24]. In a study by Tahara et al. diabetic rats which were treated by ipragliflozin had significantly lower plasma levels of IL-6, TNF-α, monocyte chemoattractant protein-1 (MCP-1), and CRP [25]. Nevertheless, it seems that the inflammation and fatty acid oxidation, which are also promoted by IL-6, have greater impact on increasing the oxidative stress in patients with T2DM. This could enhance the rate and magnitude of coronary atherosclerosis and subsequent CAD [26, 27].

## Conclusion

As EMPA-REG OUTCOME has shown beneficial effects of Empagliflozin in people with established atherosclerosis including CAD, the EMPA-CARD trial is designed to provide a better understanding of the effects of this medication on inflammation (as one of the most important mechanisms of atherosclerosis) in these patients.

## Supplementary Information

**Additional file 1.**

## Data Availability

The data/information supporting this study is available from the corresponding author upon reasonable request.

## Abbreviations

IL-6: Interleukin 6
IL-1: Interleukin 1
IL-1b: Interleukin 1-beta
CRP: C-reactive protein
hs-CRP: High sensitive C-reactive protein
GBM: Glomerular basement membrane
WBC: White blood cell
PDGF: Platelet derived growth factor
TGF-β: Transforming growth factor beta
T2DM: Type 2 diabetes mellitus
HbA_1c_: Glycated hemoglobin A_1c_
HOMA-IR: Homeostatic Model Assessment for Insulin Resistance
hs-Troponin I: High sensitive troponin I
BNP: Brain natriuretic peptide
NT-proBNP: N-terminal pro-brain natriuretic peptide
TG: Triglyceride
LDL cholesterol: Low density lipoprotein cholesterol
HDL cholesterol: High density lipoprotein cholesterol
BMI: Body mass index
eGFR: Estimated glomerular filtration rate
NYHA: New York Heart Association
SGLT2-I: Sodium-glucose co-transporter-2 inhibitor
CABG: Coronary artery bypass graft
ACS: Acute coronary syndrome
TIA: Transient ischemic attack
CVA: Cerebrovascular accident
PCI: Percutaneous coronary intervention
HTN: Hypertension
Hb: Hemoglobin
Hct: Hematocrit
DKA: Diabetic ketoacidosis
AST: Aspartate-aminotransferase
ALT: Alanine-aminotransferase
ALP: Alkaline phosphatase
Cr: Creatinine
BUN: Blood urea nitrogen
Na: Sodium
K: Potassium
PT: Prothrombin time
PTT: Partial thromboplastin time
CBC: Complete blood count
U/A: Urine analysis
U/C: Urine culture
ROS: Reactive oxygen species
MDA: Malondialdehyde
T-AOC: Total antioxidant capacity
GSHr: Reduced glutathione
CAT: Catalase
SOD: Superoxide dismutase
DCFDA: Dichlorofluorescin diacetate
FRAP: Fluorescence recovery after photobleaching
MFI: Mean Fluorescent Index
AGE: Advanced glycation end products
RAGE: Receptor for advanced glycation end products
MCP-1: Monocyte chemoattractant protein-1
TRAP-6: Thrombin Receptor Activator Peptide-6
