# Supplementary material for: Study rationale and design of a study of EMPAgliflozin’s effects in patients with type 2 diabetes mellitus and Coronary ARtery disease: the EMPA-CARD randomized controlled trial
Source: BMC Cardiovasc Disord. 2021 Jun 30;21:318. doi: 10.1186/s12872-021-02131-1 (PMC8242278; doi:10.1186/s12872-021-02131-1)
Supplement: Supplementary file 1 — Additional file 1. [file 12872_2021_2131_MOESM1_ESM.doc]

| **The assessment of EMPAgliflozin effects in type 2 diabetic patients with Coronary ARtery Disease: The EMPA-CARD Randomized Clinical Trial** | | | |
| --- | --- | --- | --- |
| **Investigator name:**  **Patient’s code:** | ______________________  ______________________ |  | **Research Council notified?** **Yes**  **No**  **What is the Recommandation ?** |

**Has the participant had any Adverse Events during this study?** **Yes**  **No *(If yes, please list all Adverse Events below)***

| **Severity** | **Study Intervention Relationship** | **Action Taken Regarding Study Intervention** | **Outcome of AE** | **Expected** | **Serious** |
| --- | --- | --- | --- | --- | --- |
| 1 = Mild  2 = Moderate  3 = Severe | 1 = Definitely related  2 = Possibly related  3 = Not related | 1 = None  2 = Discontinued permanently  3 = Discontinued temporarily  4 = Reduced Dose  5 = Increased Dose  6 = Delayed Dose | 1 = Resolved, No Sequel  2 = AE still present- no treatment  3 = AE still present-being treated  4 = Residual effects present-not treated  5 = Residual effects present- treated  6 = Death  7 = Unknown | 1 = Yes  2 = No | 1 = Yes  2 = No |

| Adverse Event | Start Date | Stop Date | Severity | Relationship to Study Treatment | Action Taken | Outcome  of AE | Expected? | Serious Adverse Event? | Initials |
| --- | --- | --- | --- | --- | --- | --- | --- | --- | --- |
| **1.** |  |  |  |  |  |  |  |  |  |
| **2.** |  |  |  |  |  |  |  |  |  |
| **3.** |  |  |  |  |  |  |  |  |  |
| **4.** |  |  |  |  |  |  |  |  |  |
| **5.** |  |  |  |  |  |  |  |  |  |
